# Associations of Oxidative Stress and Postoperative Outcome in Liver Surgery with an Outlook to Future Potential Therapeutic Options

**DOI:** 10.1155/2019/3950818

**Published:** 2019-02-13

**Authors:** Thomas Senoner, Sophie Schindler, Stefan Stättner, Dietmar Öfner, Jakob Troppmair, Florian Primavesi

**Affiliations:** ^1^Department of Visceral, Transplant and Thoracic Surgery, Medical University of Innsbruck, Innsbruck, Anichstraße 35, 6020 Innsbruck, Austria; ^2^Daniel Swarovski Research Laboratory (DSL), Department of Visceral, Transplant and Thoracic Surgery, Medical University of Innsbruck, Innsbruck, Austria

## Abstract

Several types of surgical procedures have shown to elicit an inflammatory stress response, leading to substantial cytokine production and formation of oxygen-based or nitrogen-based free radicals. Chronic liver diseases including cancers are almost always characterized by increased oxidative stress, in which hepatic surgery is likely to potentiate at least in the short term and hereby furthermore impair the hepatic redox state. During liver resection, intermittent inflow occlusion is commonly applied to prevent excessive blood loss but resulting ischemia and reperfusion of the liver have been linked to increased oxidative stress, leading to impairment of cell functions and subsequent cell death. In the field of liver transplantation, ischemia/reperfusion injury has extensively been investigated in the last decades and has recently been in the scientific focus again due to increased use of marginal donor organs and new machine perfusion concepts. Therefore, given the intriguing role of oxidative stress in the pathogenesis of numerous diseases and in the perioperative setting, the interest for a therapeutic antioxidative agent has been present for several years. This review is aimed at giving an introduction to oxidative stress in surgical procedures in general and then examines the role of oxidative stress in liver surgery in particular, discussing both transplantation and resection. Results from studies in the animal and human settings are included. Finally, potential therapeutic agents that might be beneficial in reducing the burden of oxidative stress in hepatic diseases and during surgery are presented. While there is compelling evidence from animal models and a limited number of clinical studies showing that oxidative stress plays a major role in both liver resection and transplantation and several recent studies have suggested a potential for antioxidative treatment in chronic liver disease (e.g., steatosis), the search for effective antioxidants in the field of liver surgery is still ongoing.

## 1. Introduction

A variety of inflammatory, metabolic, and proliferative liver diseases have been shown to be caused, at least partially, by redox reactions [1–8]. Reactive oxygen species (ROS) comprise oxygen free radicals, such as superoxide, hydroxyl radicals, and peroxyl radicals, with the addition of nonradicals, such as hydrogen peroxide, hypochlorous acid, and ozone [9]. In most cell types, the lion's share of intracellular oxidant production occurs in mitochondria; other important sources are nicotinamide adenine dinucleotide phosphate (NADPH) oxidases (summarized as NOX enzymes). Beyond that, a wide range of other enzymes such as nitric oxide synthase, cyclooxygenases, cytochrome P450 enzymes, xanthine oxidase, and lipoxygenases as well as other cell organelles like the peroxisome and endoplasmic reticulum contributes to ROS production [10]. Cellular structures that are primarily affected by ROS and reactive nitrogen species are proteins, lipids, and DNA. The generation of molecular oxygen in the form of reactive oxygen species is a natural part of aerobic life, where low level amounts of ROS are indispensable for the manifestation of cellular functions, such as signal transduction pathways, defense against invading microorganisms right up to gene expression, and promotion of growth or death [11, 12]. Despite this essential importance of redox reactions, excessive amounts of ROS are highly cytotoxic. The body disposes of protective measures against ROS via enzymes (e.g., superoxide dismutase (SOD), catalase (CAT), and glutathione peroxidase (GSH-Px)) as well as nonenzymatic compounds (e.g., tocopherol/vitamin E, beta-carotene, ascorbate, and glutathione (GSH)).

**Figure 1:**
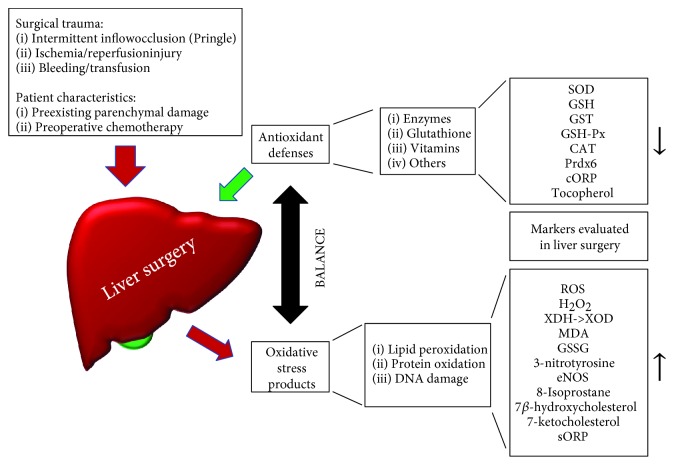
Factors influencing oxidative stress in liver surgery and biomarkers evaluated in the literature. CAT: catalase; cORP: capacity oxidative reduction potential; eNOS: endothelial nitric oxide synthase; GSH: glutathione; GSH-Px: glutathione peroxidase; GSSG: oxidized glutathione disulfide; GST: glutathione transferase; MDA: malondialdehyde; Prdx6: peroxiredoxin 6; ROS: reactive oxygen species; SOD: superoxide dismutase; sORP: static oxidative reduction potential; XDH: xanthine dehydrogenase; XOD: xanthine oxidase.

Ischemia/reperfusion (I/R) injury in the context of liver surgery has been shown to cause production of free radicals after reoxygenation of the liver, leading to lipid peroxidation and hepatocellular injury [13]. In particular, during major liver resection, intraoperative (intermittent) occlusion of the liver inflow blood supply (commonly referred to as the “Pringle maneuver”) to avoid excessive blood loss has been shown to exert significant oxidative stress in human hepatocytes, with the effect being amplified with increased duration of clamping [14]. During liver transplantation, hepatic cold and warm ischemia followed by reperfusion is a major clinical problem and it also represents an important factor which determines the postoperative outcome. Ultimately, a balance between oxidant and antioxidant agents is paramount, since too much ROS has been shown to be detrimental to the donor organ. We subsequently elucidate the role of oxidative stress in liver surgery including transplantation as well as resection (Figure 1) and discuss possible therapeutic antioxidant agents.

## 2. The Role of Oxidative Stress in Surgical Procedures: Implications for Liver Surgery in General

Oxidative stress plays an important role during different steps of many surgical procedures, such as transplantation (heart, liver, etc.), clamping/unclamping of the aorta (thoracic surgery and abdominal aortic surgery), release of a limb tourniquet (orthopedic surgery), and intermittent inflow occlusion (hepatic and cardiac surgery) [15, 16]. Even without major vessel involvement, surgery itself can cause significant physiological stress in the body and the relevance of ROS production has also been shown in general surgical procedures such as bowel resection [17]. However, in this setting, ROS levels might be reduced through application of Propofol-based anesthesia or laparoscopic techniques. Additionally, elderly patients are of particular interest, since their production of ROS is generally increased and antioxidant defenses are reduced [15].

When looking at resection for liver diseases in particular, hepatobiliary surgery has undergone tremendous advances within the last decades [18]. Previously, the limits of resectability were mainly determined by high postoperative mortality due to technical and perioperative limitations. Nowadays, after a myriad of surgical and anesthesiological advancements have led to much improved outcomes, the boundaries of liver surgery are primarily set by the volume of the remaining liver (the future liver remnant (FLR)). Given that the liver has the potential to regenerate quickly after resection, usually, a FLR of about 30% (depending on the parenchymal quality and comorbidities) is sufficient to maintain the numerous functions in the human body, including detoxification, hormone secretion, protein synthesis, and glycogen metabolism. Thus, liver surgery is an attractive therapeutic option with potential for cure in a wide range of various primary hepatic and metastatic cancerous diseases. However, as a consequence of development of more advanced procedures with the intent to broaden indications, more than 10% of patients still develop postoperative liver failure (POLF) after large resections. The association with other systemic and inflammatory complications and the lack of specific treatment for POLF remains a major problem [19]. The main cofactors leading to POLF include the amount of surgical trauma, extensive blood loss, preexisting liver parenchymal damage (steatosis, fibrosis, and cirrhosis), a too small (for patient) size FLR, and perioperative infectious complications. As depicted in Figure 1, these factors may possibly also influence perioperative, oxidative stress levels within the liver, hereby potentially affecting the capability of the patients' organism to cope with POLF and other complications.

An important technical part during liver resection is vascular inflow occlusion (VIO), which may be applied unselectively through the Pringle maneuver with a tourniquet placed around the hepatoduodenal ligament or through selective occlusion of portal vein or hepatic artery branches. This serves to reduce intraoperative blood loss and the consequent need for transfusions, which have independently been shown to increase postoperative morbidity and mortality [20]. However, the transient blockage of blood supply and subsequent reperfusion of the liver parenchyma leads to I/R injury. Interestingly, the reperfusion period appears to cause more damage than the ischemic period.

In general, two forms of hepatic ischemia can be distinguished, namely, warm and cold ischemia, having different effects on I/R injury. Warm ischemia occurs due to an interruption of hepatic blood supply, such as during transplantation, liver resection, trauma, and shock. Cold ischemia occurs during organ preservation prior to transplantation [21]. Hepatocytes are more sensitive to warm ischemia as opposed to cold ischemia, and most hepatocytes remain viable up to 48 h of cold preservation and reperfusion. Cold ischemia profoundly impairs several key functions of hepatocytes, contributing to preservation injury of the liver graft and compromised hepatic functions after liver transplantation [22]. Oxidative stress seems to play a major role in eliciting signaling pathways that lead to the onset of necrosis/apoptosis during I/R injury [8]. Following reoxygenation, ROS inflict direct tissue damage and initiate a cascade of deleterious cellular responses leading to inflammation, cell death, and hepatic failure: The first few hours after reperfusion are characterized by increased oxidative stress and represent the initial phase of I/R injury. Excessive ROS bind and alter cellular macromolecules (including DNA, proteins, and lipids), thus affecting their function, primarily in hepatocytes but also in other cell types along the vascular wall such as Kupffer cells (KC) and endothelial cells [23]. Hepatocytes mainly undergo necrosis more than apoptosis resulting in detrimental consequences due to release of cytosolic damage-associated molecular patterns (DAMP), activation of KC, and amplification of inflammatory signaling through cytokines and chemokines and also endothelial injury. Immune cells (neutrophils and monocytes/macrophages) migrate to the liver and, when activated, constitute the primary source of extracellular ROS, leading to exacerbation of oxidative stress, hepatocellular damage, and clinically relevant liver dysfunction. This late phase of injury (between 6 and 24 hours after reperfusion) characterized by neutrophil activation, further ROS release, TNF-*α*-induced expression of adhesion molecules on vascular cells, and chemokine stimulation results in exponentiation of initial damage. Inducible nitric oxide synthase (iNOS) is being upregulated, creating large quantities of nitric oxide (NO), leading to further creation of reactive nitrogen species [8]. It is important to emphasize, however, that the injurious effects of ROS are not limited to the liver. There is evidence indicating that the release of ROS from the liver into the bloodstream is primarily responsible for the systemic complications encountered after liver surgery [8].

Regarding hepatic antioxidant defense mechanisms, several endogenous enzymatic compounds acting as scavengers of ROS have been studied [24]. For example, SOD catalyzes reduction of superoxide anions to hydrogen peroxide and enhanced enzyme activity has been shown in patients' plasma after liver transplantation. Furthermore, GSH-PX found in the cytosol and mitochondria plays a major role in ROS control, with higher enzyme activity compensating for increased lipid peroxidation [25]. Catalase, an enzyme mainly located in peroxisomes, removes H_2_O_2_. Finally, peroxiredoxins reduce hydrogen peroxide to H_2_O [24].

Moreover, the liver also plays a central immunological role and hepatectomies are associated with a perpetuated inflammatory response, hereby potentially reducing the oncological outcome following surgery by contributing to relative immunosuppression [26]. Regarding the possible long-term effects of oxidative stress, ROS can cause DNA damage, leading to the accumulation of cancer-related gene mutations, which combined with histological fibrosis is a recognized risk factor for hepatocellular carcinoma (HCC) [27]. Furthermore, interleukin-17 (IL-17) plays an important role in the propagation of oxidative stress and its receptor is widely distributed on the surface of liver cells. In a mouse model, inhibiting IL-17-related pathways has been shown to effectively prevent the development of nonalcoholic steatohepatitis (NASH). In an oncological context, increased expression of serum IL-17 in patients undergoing liver surgery also correlates with a higher risk of early recurrence of HCC [28]. Additionally, there is increased evidence that surgery-induced ROS plays a key role in the development of local recurrence and distant liver metastasis following resection of other tumor entities such as colorectal cancer: More than one third of these patients develop hepatic metastases in the course of the disease, and up to half of those undergoing liver resection experience recurrence within two years. In animal models, it has been shown that an increase in severity of the surgical trauma is associated with the risk of recurrence of colorectal cancer [29]. In summary, ROS is ambivalently involved in regulation of expression of genes linked to cancer cell growth and survival, angiogenesis, invasion, and metastasis but also plays a role as an important inhibitor of cancer cell proliferation and induces apoptosis. The latter phenomenon has been extensively exploited in cancer therapy through the development of drugs capable of inducing ROS [30].

## 3. Oxidative Stress in Liver Transplantation

The vast majority of knowledge regarding oxidative stress in the setting of liver transplantation has been gathered from animal models, while evidence from human studies is limited. All available publications utilizing animal liver transplant models are presented in Table 1, while human studies are listed in Table 2. For readability reasons, first, a short synopsis of findings generated in these studies will be reported, followed by presentation of exemplary publications in detail. In summary, it has been shown that orthotopic liver transplantation (OLT) leads to a significant amount of directly measurable ROS as well as downstream markers of lipid peroxidation (malondialdehyde (MDA) and isoprostanes) and consumption of antioxidative enzymatic (SOD, CAT, and GSH) and nonenzymatic compounds (tocopherol) [31–35]. While these findings were pronounced after prolonged exposure to ischemia [36] and in obese animals with steatotic livers [31], the effect could be attenuated in models with ischemic preconditioning [31, 32] or with injection of gadolinium chloride possibly promoting antioxidant defense mechanisms [35].

In the clinical setting, it has been shown that increased preoperative levels of oxysterols in the blood or urine may be helpful to assess the individual patients' oxidative stress status and are associated with poor postoperative graft function [37, 38]. During reperfusion of engrafted livers, a significant increase in oxidative stress is seen, consistently with ischemia/reperfusion injury. Intriguingly, although these levels decrease immediately within the first days postoperatively; results on long-term levels are conflicting: In one study [37], urinary isoprostanes measured 6 to 12 months after transplantation were still significantly increased compared to normal values, while another report [39] interestingly showed not only lower MDA levels > 12 months after transplantation compared to those of healthy volunteers but also a concurrent decrease in GSH. Although the underlying cause remains unclear, two hypotheses are postulated, including interfering effects of gradual corticosteroid withdrawal and modification of other immunosuppressants or influence by recurrence of underlying liver disease. Interestingly, occurrence of acute rejection episodes, allograft HCV reinfection, or other composite adverse events showed no alterations in oxidative stress levels within the first postoperative year.

### 3.1. Animal Data

He et al. [32] assessed the impact of ischemic preconditioning in liver transplantation-induced I/R injury in rats. They randomly assigned 35 rats to four groups: a sham group, an OLT group, an ischemic postconditioning (IPostC) group, and a remote ischemic preconditioning (RIPerC) group. In the IPostC model, six 10-second cycles of reperfusion and reocclusion of the portal vein were applied immediately after inflow reperfusion. The RIPerC model consisted of three cycles of 5-minute clamping of the femoral vessels each followed by 5 minutes of reperfusion immediately at onset of the anhepatic phase. ALT, AST, Creatinine, CK-MB, and other parameters for hepatocellular damage were all significantly decreased in the RIPerC group compared with the OLT group. Markers of acute damage of the liver, kidney, and heart, on the other hand, were significantly elevated compared with those of the sham group. Levels of ROS and hydrogen peroxide were significantly increased in the OLT group compared with the sham group, whereas they were less increased in the RIPerC group. Compared with the OLT group, the IPostC group displayed significantly lower levels of ALT, AST, ROS, hydrogen peroxide, and 3-nitrotyrosine. This study demonstrated that RIPerC is similar to or better than IPostC in preventing liver graft dysfunction and apoptosis induced by hepatic I/R injury. The RIPerC group was also associated with reduced levels of Creatinine and CK-MB as opposed to the OLT group, indicating protection of other organs. The reduced plasma aminotransferase levels observed in both the IPostC and RIPerC groups point to a protective effect of remote ischemic preconditioning. Furthermore, the procedure of RIPerC is simple to perform and is possibly applicable in clinical practice.

Hori et al. [33] investigated oxidative stress-mediated damage and the behaviour of extracellular matrices in different rat models after liver surgery and transplantation. 40 rats were divided into four groups according to the surgery performed: laparotomy only (control), 60% hepatectomy, OLT with whole-liver graft, and split OLT (SOLT) with 40% small-for-size graft. All rats in the laparotomy and OLT groups survived; in contrast, the 60% hepatectomy group and especially the SOLT group showed a significantly poorer survival. Compared to the control group, the other groups showed significantly increased graft damage scores. Postoperative AST and ALT levels were all significantly increased compared to those of the control group, with the highest levels shown in the SOLT group. MDA, as a marker of oxidative stress, was also significantly increased compared to that of the control group, again with the SOLT group displaying the highest levels. Other markers of oxidative stress were also all significantly increased versus those of the control group. Phosphatidylinositol 3-kinase (PI3K) and Akt, two enzymes involved in cellular processes such as apoptosis, were not significantly different between the control and OLT groups, but there were significant differences in the 60% hepatectomy and SOLT groups versus the control group. Antioxidant enzymes (SOD and CAT) were not significantly different in the four groups. The SOLT group showed the poorest survival, which can be attributed to the small graft size combined with a dual damage, namely, shear stress with portal hypertension and I/R injury. The 100% OLT group showed good survival, but I/R injury was still observed by histopathological and biochemical findings. The 60% hepatectomy and 40% SOLT groups, both accompanied by shear stress and portal hypertension, had decreased PI3K and Akt levels, suggesting that an apoptotic process was triggered in these groups. Consequently, the inhibition of apoptotic induction due to oxidative stress via the Akt/PI3K pathway may be key to improving postoperative outcomes.

### 3.2. Human Data

Liver cirrhosis is associated with an increased oxidative imbalance, leading to an enhanced production of ROS and a reduction in the bioavailability of antioxidants. Liver transplant recipients are particularly at increased risk of oxidative stress due to preexisting hepatic impairment, intraoperative I/R injury, postoperative immunosuppression, and possible graft rejection. Augusto et al. [39] evaluated oxidative stress in healthy controls, patients with liver cirrhosis on the transplant list, and subjects who had already been transplanted. Sixty adult male patients were subdivided into 3 groups: a control group (*n* = 15), a cirrhosis group (*n* = 15), and a transplant group (*n* = 30). Biomarkers of oxidative stress that have been measured through plasma concentrations comprised of GSH, MDA, and vitamin E. Plasma GSH and MDA levels were both significantly lower in the transplant group compared to the other two groups. No significant difference was observed in the GSH and MDA plasma levels between the control and cirrhotic groups. Both the cirrhotic and transplant groups displayed similarly low vitamin E levels, compared to the control group. This study demonstrates that the late postoperative stage of liver transplantation is associated with a reduction in lipid peroxidation, as is being expressed by a decreased concentration of MDA. A reduced level of plasma GSH and vitamin E compared with healthy control subjects indicates that the oxidative balance is not achieved postoperatively. The decreased levels of plasma GSH observed in the transplant group can likely be explained by the immunosuppressive therapy that transplanted patients are treated with. Immunosuppressive agents contribute to the alteration of biomarkers of oxidative stress, as they have been found to increase the production of oxidative stress, which can lead to depletion of GSH [40].

Peroxiredoxin 6 (Prdx6) has been shown to protect cells against liver damage induced by oxidative stress. Tu et al. [36] investigated the role of Prdx6 in ischemia- and hypoxia-induced liver damage in livers of donors after brain death (DBD). DBD liver transplants have more graft complications, and acute rejections occur more frequently due to massive release of catecholamines and inflammatory mediators, leading to oxidative stress and the activation of proapoptotic caspases. Liver tissue samples from 10 DBD transplant recipients and six patients that had undergone hemangioma surgery (control group) were collected. As compared to that of the control group, the expression of Prdx6 in DBD liver tissue was significantly reduced and the NF-*κ*B signaling pathway was activated. Normal human liver cells were cultured under ischemic-hypoxic conditions to mimic brain death conditions. Levels of AST, ALT, and LDH significantly increased, and cell viability significantly decreased as cell exposure time to DBD conditions increased. Intracellular ROS levels also increased with prolonged exposure time in human liver cells, suggesting that cell damage occurs in a time-dependent manner. The activation of the NF-*κ*B signaling pathway demonstrates a potential stress-preventive role of Prdx6 in DBD liver cells being exposed to an ischemic and hypoxic environment and indicates that Prdx6 may be regulated by NF-*κ*B. Liver cells who overexpressed Prdx6 exhibited significantly reduced levels of intracellular ROS when exposed to ischemia and hypoxia. Furthermore, cell viability significantly increased in these cells. These results indicate that Prdx6 has a protective role when liver cells are exposed to ischemia and hypoxia. Thus, Prdx6 may be a novel target to alleviate liver damage in DBD and consequently improve donor liver quality.

## 4. Oxidative Stress in Liver Resection

Similar to the available evidence in the field of liver transplantation, most data regarding oxidative stress in liver resections is derived from animal models (Table 3). In summary, markers of ROS (oxidative reduction potential), lipid peroxidation (MDA), and antioxidant compounds (GSH, SOD, and Catalase) have so far been evaluated in the animal resection setting [41–46]. Also, pretreatment with Baicalein or dimethyl sulfoxide [47] increased glutathione peroxidase activity, revealing a potential for future therapeutic studies. In the human clinical setting (Table 4), the only two available studies demonstrated that patients undergoing liver resection experience an increase in markers of oxidative stress and inflammation during surgery. Intriguingly, an early postoperative increase of static oxidative reduction potential is associated with postoperative morbidity rates [26], theoretically showing future potential for early detection of ROS-associated complications. However, to date, no trial has investigated the capability of agents with antioxidative effects to prevent or treat complications in patients undergoing liver resections so far.

### 4.1. Animal Data

Adult hepatocytes are usually quiescent but have the potential to replicate, such as after partial hepatectomy (PH). Following PH, a rapid enlargement of the FLR commonly occurs to restore hepatic mass and function [48]. Haga et al. [49] investigated the role of oxidative injury of steatotic livers in a mouse hepatectomy model. Diabetic (db/db) steatotic mice and lean littermate controls underwent two-third PH. Evident lipid accumulation was only seen in db/db mouse livers. In the first 72 h following PH, liver recovery was markedly disturbed in steatotic livers of db/db mice, whereas it occurred immediately after PH in nonsteatotic livers of control mice. However, at 7 days post-PH, the recovery of steatotic livers was equivalent to that of the control group. Oxidative stress was immediately generated only in steatotic livers, peaking within 30 minutes of PH and lasting for at least 8 h. Caspase 3, the final apoptosis-executing enzyme, was immediately activated in this group, peaking at 4 h post-PH and lasting for at least 24 h. Hepatic apoptosis increased immediately after PH and peaked at 12 h post-PH. Consequently, apoptotic cell death occurred in the steatotic model at 4 h post-PH but did not occur in the control liver. Compared with those of the control group, both Fas and Fas ligand (FasL), which play a role in apoptosis, were expressed at higher levels in steatotic livers before and after PH. Akt was expressed at equivalent levels in both model types. Antioxidant enzymes as well as antiapoptotic proteins were expressed at lower levels in steatotic livers compared with normal livers. p62/SQSTM1, an autophagosome, was expressed less only in steatotic livers. p62/SQSTM1 is negatively associated with hepatic steatosis, and its reduced expression may impair antioxidant systems. This study demonstrated that the lack of p62/SQSTM1 plays a pivotal role in post-PH acute injury of steatotic livers. Initial regeneration in these livers following PH was delayed by an immediate post-PH liver injury induced by Fas/caspase-mediated apoptosis and Fas/oxidative stress-dependent necrosis.

Riehle et al. [41] investigated the role of GSH in liver regeneration after PH in mice. Glutamate cysteine ligase (GCL), an enzyme catalyzing the rate-limiting step in GSH biosynthesis, has been shown to delay hepatocyte proliferation. Mice lacking the modifier subunit of GCL (Gclm−/−) as well as wild-type (Gclm+/+) mice have been used in this study. An increase in hepatic GSH levels in the regenerating rat liver following PH has been previously reported. Therefore, the authors hypothesized that Gclm−/− mice would not show an increase in GSH after PH. GCL activity in Gclm−/− mice was less than 25% of that in wild-type mice. Gclm−/− mice maintained low baseline GSH levels and GCL activity during liver regeneration, whereas wild-type mice had a two-fold increase in GSH levels during the early phase of regeneration. Gclm−/− mice demonstrated an overall delay in DNA synthesis as well as a lower peak number of mitotic hepatocytes after PH when compared to wild-type animals. Activity of caspase 3 could not be detected in wild-type mice; however, there was caspase 3 activity in Gclm−/− mice, indicating increased apoptosis in the absence of adequate GSH. One would expect Gclm−/− mice to have increased oxidative stress, but it has been shown that these mice have significant compensation for their chronic lack of GSH by upregulating several antioxidant genes, enhancing antioxidant capabilities. The authors therefore do not believe that oxidative stress underlies the delay in liver regeneration in Gclm−/− mice. It might be of clinical significance that relatively common genetic polymorphisms exist in human Gclm genes, which impact GCL expression and GSH synthesis, possibly increasing the risk for several liver diseases.

Another study investigating GSH homeostasis examined the impact of 12/15-lipoxygenase (12/15-LOX) inhibition with Baicalein in preventing ROS-mediated cell death after hepatic ischemia and reperfusion in mice [47]. Baicalein is an antioxidant flavonoid contained in herbal supplements that inhibits 12/15-LOX. 12/15-LOX has been considered a relevant source of lipid peroxidation in I/R injury models, with glutathione peroxidase 4 (GPX4) being regarded as cell protective and functionally antagonistic to 12/15-LOX. A warm reversible ischemia of the right anterior, left anterior, and left posterior liver segments was induced by clamping the common supplying pedicle beginning 10 min after laparotomy. Three IRI and three corresponding sham-operated groups were analyzed (*n* = 10). Baicalein was injected intraperitoneally before laparotomy. Control groups were either treated with intraperitoneal injection of a corresponding volume of dimethyl sulfoxide (group 2) or left untreated, receiving a corresponding volume of saline intraperitoneally (group 3). Cell viability was significantly different after I/R injury between group 1 and the control groups. Cell death was significantly lowered by Baicalein administration. The activity of proapoptotic enzyme pathways, such as mitogen-activated protein kinase p44/42, poly-ADP-ribose-polymerase, caspase 3, and Jun-amino-terminal kinase, was all significantly decreased in Baicalein-treated mice compared to the control groups. Pretreatment with Baicalein resulted in a significant intrahepatic elevation of glutathione oxidation, whereas a more moderate increase was observed in group 2. Glutathione homeostasis has a direct influence on oxidative stress, with an upregulation of glutathione biosynthesis being directly associated with a decreased amount of cell death, leading to decreased liver damage after hepatic I/R injury. The authors stated that the beneficial effect of 12/15-LOX inhibition observed in this study must be a result of the ability of Baicalein to reduce oxidative stress, potentially influenced by GPX4 enhancement, further reducing ROS formation.

### 4.2. Human Data

Besides mechanical/surgical stress as well as blood loss, I/R injury seems to be the main cause of hepatic damage following liver resection. Reactive carbonyl species (RCS), such as lipid peroxidation products, are gaining great interest as a cause of oxidative stress contributing to I/R injury. RCS are neutral molecules able to diffuse through cellular membranes and can therefore cause damage at sites distant from their production site. Witort et al. [50] investigated RCSs released in the blood of humans subjected to hepatic resection. Levels of biomarkers of oxidative stress (protein carbonyl, MDA, and RCS) were almost the same before anesthesia, before and 10 min after clamping, but increased significantly after 10 min of reperfusion. The increase of RCS was higher compared with that of MDA. Free plasma RCS rapidly disappear, forming covalent adducts with plasma proteins, albumin being the main protein target and Cys34 the most reactive site. Therefore, the authors identified the chemical structures of RCS adducted to Cys34, identifying acrolein (ACR) as the main Cys34 RCS adduct. ACR-Cys34 adduct was detectable already before clamping and rose significantly during the reperfusion period. ACR reacts with the nucleophilic sites of proteins, forming covalently modified biomolecules, which are also thought to be involved in the onset and progression of many pathological conditions such as cardiovascular and neurodegenerative diseases. This study demonstrated that ACR is the main RCS formed during liver reperfusion in humans. Consequently, ACR represents a new target for novel molecular treatments which are aimed at reducing hepatic I/R injury.

Schwarz et al. [26] investigated oxidative stress and the antioxidant capacity in 40 consecutive patients undergoing liver resection. Blood samples were obtained prior to surgery, pre-resection, postresection, postsurgery, and on postoperative days 1 and 3. Oxidative stress was expressed as static oxidative reduction potential (sORP) and capacity ORP (cORP). They furthermore evaluated T-helper cell (Th) 1- and Th2-specific cytokines. The primary endpoint was the correlation of intraoperative oxidative stress with postoperative outcome. The secondary endpoint was a composite endpoint of a perioperative inflammatory response as measured by Th1 and Th2 cytokines as well as other proinflammatory markers. Furthermore, morbidity and mortality within 30 days of follow-up were assessed. No significant alteration in oxidative stress response measured by sORP during and immediately after liver resection was observed. However, a substantial decrease in oxidative capacity (cORP) was detected postoperatively. Both sORP and cORP remained stable during liver resection. A low remaining antioxidant capacity correlated with older patient age. A postoperative increase of oxidative stress (sORP) exceeding 3 mV was predictive for severe complications. An increase of IL-2, IL-5, and IL-6 during surgery significantly correlated with the development of severe postoperative complications. INF-*γ*, IL-4, and GM-CSF values showed a tendency towards higher levels in patients with postoperative complications, although this was not statistically significant. This study demonstrates that patients undergoing liver resection experience an increase in markers of oxidative stress and inflammation during surgery. Antioxidant capacity remains stable during liver resection but drops postoperatively due to a consumption of antioxidant capacity to maintain oxidative stress levels within a healthy range. Patients may be screened preoperatively using markers of inflammation and oxidative stress, thus enabling the detection of those at increased risk of developing an adverse postoperative outcome. Furthermore, patients with increased intraoperative inflammatory markers might benefit from prophylactic antibiotics or closer monitoring in the ICU, although this has yet to be validated prospectively.

## 5. Possible Therapeutic Effects of Antioxidants: From Bench to Bedside

Based on the role of ROS in many pathologies, as detailed above for the liver, interest for application of natural antioxidants and development of chemical antioxidative agents to ameliorate or prevent diseases has been present for several decades. The following chapter summarizes available evidence concerning antioxidant agents for the prevention and therapy of oxidative stress in different hepatic diseases (Table 5) and shows possible implications for future application perspectives in the field of liver surgery. These agents may be of special interest in in patients with increased perioperative risk such as those with steatotic or cirrhotic livers.

### 5.1. Vitamins for the Treatment of Liver Diseases

Several vitamins possessing antioxidant properties, such as vitamins C and E, have been investigated for their role as protective agents in liver diseases. Vitamin E is the major fat-soluble chain-breaking antioxidant found in the human body, and it has been regarded as one of the most potent antioxidants in nature [51]. Its biological activity goes beyond antioxidant properties and includes also the regulation of gene expression, inflammatory response, membrane-bound enzymes, modulation of cellular signaling, and cell proliferation. Furthermore, vitamin E exerts direct and indirect effects on several enzymes involved in signal transduction (e.g., protein kinase C; protein phosphatase 2A; protein tyrosine phosphatase; protein tyrosine kinase; 5-, 12-, and 15-lipoxygenases (5-, 12-, and 15-LOX); phospholipase A2 (PLA2); cyclooxygenase-2 (COX-2); and mitogen-activated protein kinase (MAPK)), all of which are unrelated to antioxidant actions [52].

Nonalcoholic fatty liver disease (NAFLD) is defined as lipid accumulation of more than 5% of hepatocytes, in the absence of other causes of liver disease such as excessive alcohol consumption or viral infections. It represents the most common chronic liver disease worldwide, and the histological spectrum ranges from simple, nonprogressive steatosis to NASH, which may progress to cirrhosis and hepatocellular carcinoma as the end stage of the disease [53]. The pathogenesis has not yet been fully elucidated, but oxidative stress has been shown to play a crucial role in the pathogenesis of steatosis, steatohepatitis, and fibrosis. ROS are potent inducers of c-Jun N-terminal kinases (JNK), which belong to a subgroup of the MAPK superfamily. ROS activates JNK, activating a cascade of signaling pathways, which ultimately leads to apoptosis of hepatocytes [54, 55]. TNF-*α* pathway activation has also been implicated in apoptosis of hepatocytes. Furthermore, ROS inhibits VLDL secretion from hepatocytes, thus favoring steatosis [56]. Animal studies on rats fed with a methionine- and choline-deficient (MCD) diet, a widely employed NASH model, showed that vitamin E significantly reduces oxidative stress. In these rats, supplementation with vitamin E replenished hepatic glutathione; reduced steatosis, inflammation, hepatic stellate cell activation, and collagen mRNA expression; and ameliorated fibrosis [57]. Another study using an animal NASH model demonstrated that the combination of MCD diet and vitamin E significantly lowers serum transaminase levels and improves hepatic steatosis and necroinflammation. These effects are achieved through the suppression of the expression of fibrotic genes (TGF-*β* and MMP-2), COX-2, and proapoptotic genes (Bax). Furthermore, vitamin E enhances the activity of hepatic SOD and inhibits that of NF-*κ*B [58]. Several human studies have confirmed these findings regarding the benefit of vitamin E in treating NAFLD. The odds of developing hepatic steatosis in individuals with CT-diagnosed NAFLD after 4 years of active therapy were reduced with vitamin E combined with vitamin C and atorvastatin [59]. The combination of ursodeoxycholic acid (UDCA) with vitamin E improved steatosis and transaminase levels compared with either UDCA alone or placebo [60]. Vitamin E has not been shown to be beneficial in patients with diabetes, and therefore, the European Association for the Study of the Liver (EASL) and the American Association for the Study of Liver Diseases (AASLD) guidelines consider vitamin E as a potential short-term treatment for nondiabetic adults with biopsy-proven NASH [61]. There are safety concerns regarding the long-term use of vitamin E, as some meta-analysis demonstrated an increased all-cause mortality, whereas other studies suggested that vitamin E has no effect on all-cause mortality at doses up to 5500 IU/day [62–64].

### 5.2. COX Inhibitors Reduce Hepatic I/R Injury

Demiryilmaz et al. [65] investigated the effects of nimesulide on hepatic I/R injury in rats. Nimesulide is a selective COX-2 inhibitor with anti-inflammatory, analgesic, and antipyretic properties. The study consisted of 4 groups with 6 rats each: the liver ischemia reperfusion (LIR), 50 mg/kg nimesulide + liver ischemia reperfusion (NLIR-50), 100 mg/kg nimesulide + liver ischemia reperfusion (NLIR-100), and control sham operation (SG) groups. The surgical procedure consisted of clamping of the hepatic artery, portal vein, and bile duct for 1 h followed by 6 hours of reperfusion. Liver biopsies were also collected and examined biochemically (GSH and MDA levels and MPO enzyme activities). MDA liver tissue concentrations as well as MPO activity in the SG and NLIR 100 groups were nearly equal, whereas concentrations in the LIR and NLIR 50 groups were significantly higher. GSH liver tissue levels were lower in the LIR, NLIR 50, and NLIR 100 groups compared to the SG group. These results show that 100 mg/kg nimesulide almost entirely prevented oxidative stress induced through ischemia/reperfusion. Endogenous antioxidants such as GSH decreased significantly less with the higher nimesulide dosage compared to those of the other groups, whereas the oxidant parameters MDA and MPO increased significantly less with nimesulide 100 mg/kg. Consequently, nimesulide may be beneficial in the prevention/treatment of hepatic I/R injury.

### 5.3. Resveratrol, a Potential Therapeutic Agent in NAFLD

A double-blind, placebo-controlled, randomized clinical study conducted by Faghihzadeh et al. [66] investigated the effect of resveratrol on inflammatory biomarkers in NAFLD patients. Resveratrol is a natural polyphenol found in a variety of plant species which exerts beneficial effects on several illnesses such as cancer, cardiovascular disorders, diabetes, metabolic diseases, and chronic liver diseases. 50 patients were randomly assigned to two groups; one group was given a capsule containing 500 mg trans-resveratrol, and the other group received the same amount of medium-chain triglyceride as placebo, once a day for 12 weeks. Additionally, the patients were advised to follow an energy-balanced diet and perform regular physical activity. The primary endpoint was a significant reduction in ALT concentration, whereas the secondary outcome included a transient elastography score, inflammatory factor concentrations in serum and peripheral blood mononuclear cell nuclear extracts, anthropometric variables, and serum concentrations of AST, *γ*-GT, total bilirubin, and alkaline phosphatase. After 12 weeks, serum ALT and AST were significantly reduced in both groups, although a greater decrease was observed in the resveratrol group. Significant changes in inflammatory markers (hs-CRP, IL-6, and NF-*κ*B) were only observed in the resveratrol group. Cytokeratin-18 M30, a biomarker of hepatocellular apoptosis, decreased significantly in the resveratrol group compared to the placebo group. The reduction of hepatic fibrosis measured via transient elastography, however, was not significant. Hepatic tissue echogenicity decreased significantly in both groups, although a more marked reduction was observed in the resveratrol group. In this study, it was shown that additional therapy with 500 mg/d resveratrol is superior to lifestyle modification alone in the treatment of NAFLD. This beneficial effect is ascribed, at least partially, to its modulatory effect on inflammatory markers and the reduction of hepatocellular apoptosis.

### 5.4. Hyperlipidemia as a Cause of Increased Oxidative Stress

Xu et al. demonstrated that a high-fat diet is associated with increased production of reactive oxygen species, lipid peroxidation, and oxidative injury [67]. Polysaccharides extracted from *Ulva pertusa*, a green alga, have been reported to have antihyperlipidemic, antitumor, antiviral, and antioxidant activities. Qi and Sun [68] investigated the in vivo antioxidant activity of high-sulfate content polysaccharide derivative (HU) in cholesterol-rich diet-treated rats. Antioxidant activities were measured using MDA, SOD, GSH-Px, and CAT assay kits. 84 rats were randomly divided into 7 groups (*n* = 12). All rats received a cholesterol-rich diet except those in group 1 (control group). Group 2 served as hyperlipidemia control, whereas group 3 was treated with polysaccharide extracted from *U. pertusa* (U) (250 mg/kg). Groups 4, 5, and 6 were treated with HU in doses of 125, 250, and 500 mg/kg, respectively, while group 7 received cholestyramine, 500 mg/kg, serving as a positive control group. Additionally, groups 3-7 received different doses of U, HU, and cholestyramine by oral administration for 28 days. After 4 weeks, the rats were not fed for at least 12 hours, then they were weighed, and blood samples were collected. The HU-fed group (125 mg/kg) had an optimal effect on triglycerides (TG) compared to the hyperlipidemia group, but a lesser impact on total cholesterol (TC), LDL cholesterol, and HDL cholesterol. On the other hand, doses of 250 and 500 mg/kg had significant effects on TC. The antihyperlipidemic activity of 125 mg/kg HU was the strongest, compared to that of the control group, and TG were significantly decreased. This demonstrates that the antihyperlipidemic activity is not concentration dependent for HU-fed rats. Compared to the hyperlipidemia group, MDA production was decreased in the HU-fed groups. Compared to the control group, however, MDA was increased. HU inhibits the formation of MDA, leading to decreased lipid peroxidation and consequently to decreased oxidative stress. Increased MDA levels have been reported in previous studies in hyperlipidemia rats [69]. The levels of SOD and GSH-Px increased significantly with HU therapy. The treatment of hyperlipidemic rats with U and HU increased CAT activity in the liver, especially the groups receiving HU at low and middle dosages, compared to the control group. The positive group, however, showed no alteration of CAT activity compared to the hyperlipidemia group. This study demonstrated that U and HU possess antioxidant activity in vivo, in which HU exhibited stronger antioxidant activity. The authors concluded that HU protects the liver tissue from oxidative damage in cholesterol-rich diet rats through its antioxidant properties and therefore may be of use as an antihyperlipidemic agent.

### 5.5. Alpha-Lipoic Acid as a Potential Hepatoprotective Agent

Alpha-lipoic acid (ALA) is a natural protein-bound cofactor for mitochondrial *α*-ketoacid dehydrogenases synthesized in the mitochondria of plants and animal cells, which plays a major role in mitochondrial energy metabolism. In vitro studies suggest that ALA possesses potent biological antioxidant activities, scavenging reactive oxygen species and reactive nitrogen species as well as chelating redox-active metals (e.g., iron and copper). Due to these properties of ALA, Mahmoud et al. [70] investigated the role of ALA in the treatment of acetaminophen-induced liver damage in rats. 18 rats were randomly subdivided in 3 groups: a control group, which were given 1 ml of 50% propylene glycol in water orally, an acetaminophen (APAP) group receiving 3 g/kg APA in 50% propylene glycol administered orally (acute overdose), and an ALA + APAP group receiving a single 100 mg/kg dose of ALA in 0.5% alkaline saline (0.5% NaOH) orally followed by an overdose of APAP (3 g/kg) 1 h after ALA administration. All rats were sacrificed 24 hours after APAP intoxication, and the livers were removed and processed for histological and histochemical examination. H&E-stained liver sections of the control group showed normal hepatic architecture. Hepatic sections of APAP-treated rats, on the other hand, displayed centrilobular necrosis, moderate vacuolar degeneration, and inflammatory cell infiltration. The livers which had been pretreated with ALA showed fewer pathological changes compared to those of the APAP-treated group; however, normal histological structure was not fully retained. ALA protected the liver from centrilobular necrosis and inflammatory cell infiltration, though the hepatocytes were vacuolated and showed weak carbohydrate, protein, and DNA staining. This study demonstrated that ALA at a dose of 100 mg/kg produced a marginal protective effect against APAP-induced hepatotoxicity. A shortcoming of this study is certainly the inability to assess the long-term effect of ALA on the liver in acute acetaminophen toxicity. It remains to be established whether ALA has a protective effect on the liver in the long run.

### 5.6. Coffee Exhibits Antioxidant Properties

Coffee, the most consumed beverage in the world, has been shown to reduce the risk of advanced liver disease and its complications, as well as decreasing all-cause mortality. Salomone et al. [71] evaluated the pathophysiological mechanisms by which coffee exhibits beneficial effects in NAFLD. Twenty-four male rats were divided into 4 groups; 2 groups were fed a high-fat diet (HFD) for a total of 3 months and 2 groups were fed a standard diet for the same period. Starting with the second month, the two HFD groups and the two groups on a standard diet were given different beverages; one of each group drank decaffeinated coffee and the other drank water. After 3 months, all animals were sacrificed; serum and liver samples were obtained and snap frozen for further analyses. Serum ALT, total cholesterol, and triglycerides were measured. As biomarkers of lipid peroxidation and DNA damage, liver 8-isoprostanes and 8-hydroxy-2′-deoxyguanosine were measured. Additionally, whole-liver homogenates were processed for Western blot analysis. Coffee administration had no impact on food and caloric intake, as both groups receiving an HFD increased their body weight by 20% compared to rats receiving the standard diet. Serum ALT increased 3-fold in the HFD group compared to the standard diet group, whereas serum ALT was reduced to healthy control levels in the HFD group who consumed coffee. HFD led to an increase in serum triglycerides, whereas coffee decreased triglycerides. Liver triglyceride levels increased almost 4-fold in the HFD and water group compared to the standard diet group and were decreased 2-fold through the administration of coffee. HFD led to panacinar steatosis, with diffuse ballooning and foci of inflammatory cells throughout the lobule in liver sections; these features were reduced by coffee administration. Using quantitative PCR, increased gene expression of 2 endoplasmic reticulum chaperones, namely, glucose-regulated protein 78 (GPR-78) and protein disulfide-isomerase A3, was observed only in rats fed an HFD and coffee. Furthermore, the coffee group also exhibited increased hepatic expression of 2 mitochondrial chaperones, DJ-1 and mitochondrial heat stress protein 70 (mtHsp70). Additionally, reduced lipid peroxidation and DNA oxidative damage, as measured by levels of 8-isoprostanes and 8-hydroxy-2′-deoxyguanosine, were observed. Several other studies have reported an inverse association of coffee consumption and liver disease [72–74]. Coffee induces the expression of antioxidant and stress sensor proteins, particularly peroxiredoxin 1 (PRDX1), a prototypical antioxidant protein. PRDX1 inhibits the activation of JNK, which has been involved in the pathogenesis of NASH. Furthermore, PRDX1 counteracts tumorigenesis through PTEN signaling. The ability of coffee to prevent the development of hepatocellular carcinoma may be explained by the induction of the antitumor proteins mtHSP70 and PRDX1. Another antioxidant protein induced by coffee is D-dopachrome tautomerase, an enzyme involved in melanin synthesis, which has been reported to prevent experimental fibrinogenesis. Additionally, D-dopachrome tautomerase has been shown to inhibit adipogenesis and improve insulin resistance.

### 5.7. N-Acetyl-L-Cysteine

Villagarcía et al. [75] evaluated the effect of long-term N-Acetyl-L-Cysteine (NAC) in hypothalamic obese rats. The rats are characterized by prediabetes, dyslipidemia, obesity, increased inflammatory state, and liver dysmetabolism accompanied by increased oxidative stress. This is based upon the findings that the hypothalamus is mainly responsible for energy homeostasis, namely, the balance between energy intake, metabolism, storage, and expenditure. The i.p. administration of monosodium L-glutamate (MSG) in newborn rats is known to lead to the phenotype of hypothalamic obese rats. Newborn male Wistar rats were injected i.p. with MSG. Littermate controls (C) were injected i.p. with a 10% NaCl solution. At 21 days of age, rats were weaned and housed in a controlled environment. On the weaning day, rats were divided into two groups: littermate controls (C) and MSG-treated rats (MSG) which received Purina rat chow and water as desired until the experimental day. The other two groups (C and MSG rat groups) received Purina rat chow and either water or NAC solution as desired (C-NAC and MSG-NAC groups, respectively). Each group was composed of 8-10 rats. On the experimental day (150 days of age), the rats were euthanized, the trunk blood was collected, the brain was dissected to assess the effectiveness of MSG treatment by macroscopic observation of degenerated optic nerves (inclusion criteria), and the liver was dissected and weighed. Rats from all four groups displayed similar glycemic levels; however, MSG rats displayed significantly higher plasma insulin levels. NAC treatment in the latter group was fully able to decrease insulin concentrations reaching values similar to those in C rats. NAC could reduce insulin levels in C rats to levels even lower than those displayed by NAC-untreated C rats. Consequently, high homeostasis model assessment insulin resistance (HOMA-IR), *β*-cell function (HOMA-*β*), and the reduced liver insulin sensitivity index (LISI) values (markers of insulin resistance) displayed by MSG rats were all fully repealed when treated with NAC. The same effect on the aforementioned insulin indexes was observed in C rats treated with NAC. Levels of triglyceride, uric acid, AST, and ALT were all significantly higher in the MSG group compared to the C group. The increment in these blood levels in MSG rats could be fully prevented by the administration of NAC. Levels of thiobarbituric acid-reactive substances, a key marker of oxidative stress, were significantly reduced through NAC treatment both in MSG and C rats. MSG rats displayed increased levels of oxidative stress, increased content of protein carbonyl groups and iNOS in liver tissue, and concordantly reduced levels of GSH. NAC administration in these rats significantly reduced the levels of protein carbonyl groups and iNOS in liver tissue and elevated levels of GSH. The treatment of neonatal rats with MSG resulted in rats with deeply inflamed livers compared to C rats. MSG rats displayed increased levels of Il1b and PAI1 in the local tissue mRNA and increased liver protein content of TNF*α* and COX-2, all markers of increased oxidative stress. Chronic oral NAC therapy was able to prevent a rise in these levels (*P* < 0.05 vs. the MSG group). This study demonstrated that daily oral NAC treatment can prevent endocrine-metabolic disturbances and hepatic dysfunction developed by MSG rats, suggesting an increased oxidative state as the underlying cause. Insulin resistance has also been associated with increased oxidative stress, and this could be reduced in this study by the administration of NAC as assessed by HOMAs and LISI scores. The authors even suggested the coadministration of antioxidants (such as NAC) with statins to reduce comorbidities frequently encountered in diseases such as the metabolic syndrome or Cushing's syndrome.

The encouraging results on NAC preventing oxidative stress obtained in liver disease animal models, however, could not be confirmed in human studies after liver resection. A randomized clinical trial by Grendar et al. [76] analyzed the effects of NAC on liver recovery after resection in 206 patients. The patients were randomized to postoperative conventional therapy (*n* = 110) or postoperative NAC administration (*n* = 96). There were no significant differences in overall complications or hepatic failure between the two treatment groups. In fact, significantly more delirium was observed in the NAC group, causing early trial termination. Only randomization to NAC and extensive resections were predictive of postoperative complications. This study demonstrated no benefit of postoperative NAC administration, and there was even a trend toward more complications and a higher rate of delirium in the NAC group.

Therefore, NAC administration following liver resection can currently not be recommended as a therapy for preventing postoperative complications.

### 5.8. Coenzyme Q10 after Resection of Hepatocellular Carcinoma

Liver cancer has been reported by the World Health Organization (WHO) to be the second leading cause of cancer deaths worldwide [77]. HCC, which accounts for the majority of liver cancers, has been shown to be negatively influenced by high levels of oxidative stress and inflammation, which promote its progression. Coenzyme Q10, an intracellular antioxidant, is a key component of the mitochondrial respiratory chain for adenosine triphosphate synthesis. A single-blinded, randomized, placebo-controlled study conducted by Liu et al. [78] evaluated for the first time the effects of coenzyme Q10 supplementation in patients who had recently undergone resection of HCC. 41 patients were randomly assigned to the placebo group (*n* = 20) or coenzyme Q10 (Q10-300) group (*n* = 21). The patients were instructed to take two capsules daily (coenzyme Q10 supplements 150 mg/b.i.d.) for 12 weeks. The levels of coenzyme Q10, vitamin E, oxidative stress, and antioxidant enzymes were measured. As would be expected, the plasma coenzyme Q10 concentration was significantly higher in the Q10-300 group compared to the placebo group. The plasma MDA level was significantly decreased, and levels of antioxidant enzymes such as SOD, GPX, and CAT were significantly increased through coenzyme Q10 supplementation. Furthermore, the plasma coenzyme Q10 concentration significantly correlated with the levels of vitamin E and antioxidant enzyme activities; however, it did not correlate with the level of inflammatory markers. This pilot study demonstrated that Coenzyme Q10 supplementation significantly increased the antioxidant capacity and reduced oxidative stress and inflammatory levels in HCC patients postoperatively. However, the study was not powered to and did not evaluate the short-term effects on postoperative complications or the long-term effects concerning tumor recurrence or overall survival. In summary, coenzyme Q10, which has shown a significant anticarcinogenic activity in various cancer models via its antioxidant and anti-inflammatory properties, is a safe and well-tolerated supplement. Through its antioxidant and anti-inflammatory properties, coenzyme Q10 may represent a potential complementary therapeutic agent perioperatively or in chronic disease patients who suffer from an elevated status of oxidative stress or inflammation.

### 5.9. Dark Chocolate as a Therapeutic Agent in NASH

Experimental studies have shown that the activation of NADPH oxidase (NOX) represents a pathogenetic mechanism determining fibrosis and disease progression in NASH [79]. The activation of NOX isoform 2 (NOX2) has been associated with increased liver damage and plays a pivotal role in the pathogenesis of NASH. Nutrients rich in polyphenols, such as cocoa, exert antioxidant properties; dark chocolate as an example has been shown to be able to reduce oxidative stress via inhibition of the activation of NOX2 [80]. Loffredo et al. [81] investigated the relationship between NOX2 activation and NASH and the effect of dark chocolate (cocoa) on oxidative stress and liver damage in NASH-afflicted patients. At first, they assessed oxidative stress by measuring blood levels of isoprostanes and soluble NOX2 (sNOX2-dp), a marker of NOX2 activation, as well as hepatocyte apoptosis, assessed by serum levels of CK-18, in 19 NASH patients and 19 matched controls. Thereafter, NASH patients were randomly assigned to a treatment with 40 g/day of dark chocolate (≥85% cocoa) or milk chocolate (≤35% cocoa) for 2 weeks. Supplement with dark chocolate resulted in a significant increase in serum total polyphenols and epicatechin metabolite compared to baseline levels; milk chocolate, on the other hand, had no impact on the levels of polyphenols and epicatechin metabolite. Similarly, dark chocolate intake resulted in a significant reduction in sNOX2-dp, isoprostanes, and CK-18 M30 levels; however, no changes were observed after milk chocolate ingestion. Compared to baseline levels, no significant differences were found for fasting blood glucose, insulin, total cholesterol, LDL and HDL cholesterol, triglycerides, AST, and *γ*-GT for either dark or milk chocolate ingestion. The only significant difference was a decreased ALT level after 2 weeks of dark chocolate intake. In NASH patients, NOX2 is significantly upregulated and dark chocolate intake has been shown in this study to reduce serum levels of sNOX2-dp, isoprostanes, and CK-18. NOX2-generated oxidative stress is associated with the severity of ultrasound liver steatosis; targeting NOX2 may therefore represent a novel therapeutic approach to slow liver disease progression. Polyphenols contained in cocoa could potentially be a useful therapy to reduce oxidative stress in NASH through their antioxidant activity via NOX2 downregulation. Limitations of the study are the small sample size and the short therapeutic duration; therefore, further studies are needed to assess the therapeutic benefits of dark chocolate in NASH patients.

## 6. Conclusion

In summary, oxidative stress plays a major role in a variety of chronic liver disorders, but also in liver surgery. Especially, in liver transplantation, ischemia/reperfusion injury is accompanied with the development of ROS as has been shown by a number of animal and human studies. In the field of liver resections, a number of cofactors such as underlying liver disease (e.g., steatosis) or volume of resection have been shown to be linked with the extent of oxidative stress. However, the association of increased oxidative stress with postoperative morbidity or liver failure in the human setting has so far only been evaluated by one study, highlighting the need for further studies in this setting. Also, most evidence on antioxidative treatment comes from studies on chronic liver diseases, particularly steatosis. Interventional trials in patients undergoing liver resection or transplantation are still lacking. Since postoperative liver failure continues to represent a major problem in hepatic surgery due to shortage of effective treatment, the evaluation of a causative link between ROS and liver failure and consecutive development of a specific, antioxidative treatment seem crucial.

## Figures and Tables

**Table 1:** Findings on the role of oxidative stress/ROS during liver transplantation (animal studies).

Reference	Year	Biomarkers	Study subjects	Results
Fernandez et al. [31]	2004	SOD, GSH, XDH + XOD, MDA	Group 1 (sham group): *n* = 12; 6 lean and 6 obese Zucker rats Group 2 (transplantation group): *n* = 24; *n* = 12, 6 transplantations with steatotic livers being flushed with cold solution for 6 h prior to transplantation; *n* = 12, 6 transplantations with rats as donors and recipients Group 3 (transplantation and preconditioning group): the same procedure as group 2 with 5 min of ischemia followed by 10 min of reperfusion prior to transplantation	SOD: an equal decrease in groups 2 and 3 was observed compared to that in the sham group; preconditioning had no effect on the decrease of SOD; GSH: decreased in group 2 but were comparable to that of the sham group in group 3 XDH + XOD: no significant change of XDH + XOD activity was witnessed, but in group 2, there was a conversion from XDH to XOD, which was stronger in obese rats; preconditioning attenuated this conversion MDA: increased in group 2 compared to that in the sham group; obese rats showed a stronger increase than lean rats; preconditioning attenuated this effect
He et al. [32]	2017	ROS, H_2_O_2_	35 Sprague-Dawley rats Group 1 (sham group): *n* = 5 Group 2 (OLT group): *n* = 5 Group 3 (IPostC group): *n* = 5, OLT with portal vein reperfusion and reocclusion for six 10-sec cycles after onset of reperfusion Group 4 (RIPerC group): *n* = 5, OLT with hindlimb ischemia and reperfusion for three 5 min cycles at the beginning of the anhepatic phase; 15 donor rats	ROS: levels of ROS were significantly elevated in the OLT group compared to sham group, IPostC and RIPerC attenuated production of ROS H_2_O_2_: levels were elevated in all groups, but lower in the sham, IPostC, and RIPerC groups compared to the OLT group
Hori et al. [33]	2014	MDA, SOD, CAT	40 Lewis rats (*n* = 10 each) Group 1 (control) Group 2 (60% hepatectomy) Group 3 (OLT) Group 4 (40% split OLT)	MDA: group 1 < groups 2, 3, and 4; group 2 < group3 < group 4 SOD: group 1 > groups 2, 3, and 4; group 2 = group3 = group 4 CAT: group 1 > groups 2, 3, and 4; group 2 = group3 = group 4
Ngo et al. [34]	2013	MDA, tGSH	45 Sprague-Dawley rats (*n* = 9 each) Group 1 (isogenic control group): liver perfused with Sprague-Dawley rat whole blood Group 2 (erythrocyte group): liver perfused with human erythrocytes and serum Group 3 (thrombocyte group): liver perfused with human thrombocytes, erythrocytes, and serum Group 4 (KC-group): liver perfused with human whole blood; KC depleted before reperfusion with GdCl_3_ Group 5 (xenogeneic whole-blood group): liver perfused with human whole blood	MDA: plasma levels significantly elevated in the xenogeneic whole blood group and in the KC group, but to a lower extent; plasma levels in all other groups are not significantly elevated tGSH: tGSH levels fell in groups 1, 2, 3, and 5 and slowly recovered after reperfusion, although not to basal values; between groups 1, 2, and 3, there were no significant differences in tGSH levels; levels in group 5 showed the strongest decline in tGSH levels; levels in the KC group remained at basal levels
Schauer et al. [35]	2001	GSH, GSSG	22 Lewis rats; Group 1 (treatment group; *n* = 8): administration of GdCl_3_ 48 hours and 24 hours prior to harvesting Group 2 (untreated group; *n* = 8): injection of identical volumes of saline at the same timepoints as group 1 Group 3 (sham group; *n* = 6)	GSH: plasma levels elevated in groups 1 and 2 until the end of reperfusion but remained unchanged in the sham group GSSG: plasma levels strongly elevated in group 2; GdCl_3_ pretreatment attenuated this elevation in group 1; no changes in the sham group

CAT: catalase; GdCl_3:_ gadolinium chloride; GSH: glutathione; GSSG: oxidized glutathione disulfide; IPostC: ischemic postconditioning; KC: Kupffer cell; MDA: malondialdehyde; OLT: orthotopic liver transplantation; RIPerC: remote ischemic preconditioning; ROS: reactive oxygen species; tGSH: total glutathione; SOD: superoxide dismutase; XDH: xanthine dehydrogenase; XOD: xanthine oxidase.

**Table 2:** Studies evaluating oxidative stress/ROS during liver transplantation in humans.

Reference	Year	Biomarkers	Study subjects	Results
Augusto et al. [39]	2014	rGSH, MDA, Vit. E	60 male patients between 27 and 67 Group 1 (control) = 15 healthy volunteers Group 2 (cirrhosis) = 15 Group 3 (transplantation) = 30	rGSH: group 1 > group 2 > group 3 MDA: group 1 = group 2; group 3 < group 1 and group 3 Vit. E: group 1 > group 2 and group 3; group 2 = group 3
Burke et al. [37]	2002	Urinary dinor-dihydro iPF_2*α*_-III levels	50 patients undergoing orthotopic liver transplantation	Dinor-dihydro iPF_2*α*_-III levels were elevated presurgical, rose significantly after reperfusion, became low on post-OP day 4, but never reached the levels of the healthy control group
Corradini et al. [38]	2005	7*β*-Hydroxycholesterol, 7-ketocholesterol	32 patients with OLT	Preoperative high 7*β*-hydroxycholesterol and 7-ketocholesterol levels were predictive for initial poor graft function (IPGF); postoperative increases of 7*β*-hydroxycholesterol and 7-ketocholesterol were nonsignificant
Tu et al. [36]	2015	Prdx6, ROS	10 DBD patients 6 patients undergoing hemangioma surgery (=control)	Prdx6: DBD patients < control ROS: intracellular ROS levels increase with a time of exposure to ischemia

DBD: donors after brain death; IPGF: initial poor graft function; MDA: malondialdehyde; OLT: orthotopic liver transplantation; Prdx6: peroxiredoxin 6; rGSH: reduced glutathione; ROS: reactive oxygen species.

**Table 3:** Studies investigating the role of oxidative stress/ROS during liver resection (animal studies).

Reference	Year	Biomarkers	Study subjects	Results
Battal et al. [42]	2015	MDA, GSH, GST, GSH-Px, SOD	24 Wistar albino rats Group 1 = 8 controls Group 2 = 8 with 70% hepatectomy Group 3 = 8 with 70% hepatectomy + subsequent administration of GLP-1 analogue 2 times a day	MDA: group 1 ↓, group 2 ↑, group 3 < group 2 GSH, GST, GSH-Px, SOD: group 1 ↑, group 2 ↓, group 3 > group 2
Beyer et al. [43]	2008	Dihydroethidine (fluorescent dye that intercalates into DNA upon oxidation)	NRF2 knockout mice (reduced cytoprotective enzymes resulting in oxidative stress) versus wild-type mice	NRF2 knockout mice showed stronger nuclear fluorescence than wild-type animals
Drefs et al. [47]	2017	GSSG/GSH as indirect measure of glutathione peroxidase activity	60 male C57BL/6 wild-type mice divided into 3 I/R groups (60 min of ischemia, 90 min reperfusion) and 3 corresponding sham groups Baicalein administered intraperitoneally 30 minutes before laparotomy; controls received an equal volume of either the vehicle (DMSO) or saline	Pretreatment with Baicalein increased intrahepatic glutathione peroxidase activity significantly; sole application of DMSO also elevated the activity level, although not statistically significant
Florholmen-Kjær et al. [44]	2016	GSH	12 pigs: 6 PH_x_ with 60% hepatectomy, 6 controls	GSH PH_x_ = control
Haga et al. [49]	2014	roGFP	Male homozygous db/db mice Lean mice (=control)	OS occurred in db/db mice immediately after PH, peaked at 30 minutes, and remained elevated for at least 8 hours; a significant difference between controls and db/db mice was shown
Hori et al. [33]	2014	MDA, SOD, CAT	40 Lewis rats Group 1 = 10 controls Group 2 = 10 60% hepatectomy Group 3 = orthotopic liver transplant Group 4 = 40% split orthotopic liver transplant	MDA: group 1 < groups 2, 3, and 4; group 2 < group 3 < group 4 SOD: group 1 > groups 2, 3, and 4; group 2 = group 3 = group 4 Catalase: group 1 > groups 2, 3, and 4; group 2 = group 3 = group 4
Mosbah et al. [45]	2014	MDA, SOD, CAT	25 male Sprague-Dawley rats Sham group = 5 mobilization but no hepatectomy; PH = 5 standard hepatectomy without prior mobilization; NCPH = 5 protocol like sham with following hepatectomy; TCPH = 5 clamping of the entire hepatic pedicle (2 × 10 min ischemia, 5 min reperfusion) with following hepatectomy; RPH = 5 lobes to be resected were mobilized and clamped (2 × 10 min ischemia, 5 min reperfusion) with following hepatectomy	MDA: after hepatectomy ↑ in PH, NCPH, TCPH, and RPH compared with the sham group; TCPH > PH > NCPH, all with peaks at 24 h; RPH also increased but without a peak and with a faster decrease SOD and catalase: levels decreased in all groups compared to the sham group; the lowest levels are found in TCPH and the highest levels in RPH
Riehle et al. [41]	2014	GSH	Gclm−/− and Gclm+/+ (wild-type = control) mice 2/3 PH was performed on 8-10-week-old males	Baseline GSH levels of Gclm−/− mice are about 15% of wt mice and Gclm−/− mice maintained low GSH levels after 2/3 PH, whereas wt mice showed a 2-fold increase of GSH levels during the early phase of regeneration Gclm−/− mice showed a delayed peak in cell proliferation compared to wt mice
Saito et al. [46]	2014	MDA, SOD, CAT, GSH-PH_x_	50 Wistar rats Group 1 = 25 with GTE + 90% hepatectomy pretreatment Group 2 = 25 without GTE pretreatment + 90% hepatectomy	MDA: group 1 < group 2, equal by day 7 SOD, CAT, GSH-PH_x_: group 1 > group 2, SOD is equal by day 7

CAT: catalase; DMSO: dimethyl sulfoxide; Gclm: glutamate cysteine ligase; GSH: glutathione; GSH-Px: glutathione peroxidase; GSSG: oxidized glutathione disulfide; GST: glutathione transferase; GTE: green tea extract; MDA: malondialdehyde; PH: partial hepatectomy; roGFP: reduction-oxidation-sensitive green fluorescent protein; SOD: superoxide dismutase.

**Table 4:** Publications examining oxidative stress/ROS during liver resection in humans.

Reference	Year	Biomarkers	Study subjects	Results
Schwarz et al. [26]	2017	sORP, cORP	40 patients undergoing elective hepatectomy > 2 segments	sORP: no significant changes during and after hepatectomy; cORP: stable perioperatively, significantly decreases postoperatively and on post-OP days 1 and 3; increase of sORP >3 mV post-OP predictive for severe complications
Witort et al. [50]	2016	MDA	6 patients undergoing PH for hepatic metastases	MDA unchanged during administration of anesthesia until 10 minutes after reperfusion, and then increased

cORP: capacity oxidative reduction potential; MDA: malondialdehyde; PH: partial hepatectomy; sORP: static oxidative reduction potential.

**Table 5:** Agents investigated regarding their potential therapeutic antioxidant effects.

Study reference	Antioxidant agent(s)	Studied population	Main therapeutic antioxidant effects
[57–61]	Vitamins C and E	Animal and human studies	↓ oxidative stress, steatosis, inflammation, hepatic stellate cell activation, collagen mRNA expression, fibrosis ↑ hepatic glutathione Not beneficial in diabetics
[65]	COX inhibitors (nimesulide)	Animal studies	Reduced GSH decreases and MDA and MPO increase with nimesulide, reducing hepatic I/R injury
[66]	Resveratrol	Human studies	↓ ALT and AST, inflammatory markers (hs-CRP, IL-6, and NF-*κ*B), cytokeratin-18 M30 in NAFLD
[68]	Polysaccharides extracted from *Ulva pertusa*	Animal studies	Antihyperlipidemic effect (↓ TG, TC, LDL cholesterol , HDL cholesterol) ↓ MDA ↑ SOD, GSH-Px, CAT
[70]	Alpha-lipoic acid	Animal studies	Preserves normal hepatic architecture shortly after acetaminophen-induced liver damage
[71–74]	Coffee	Animal and human studies	↓ ALT, triglycerides, lipid peroxidation, and DNA oxidative damage; counteracts tumorigenesis; inhibits adipogenesis; improves insulin resistance
[75, 76]	N-Acetyl-L-Cysteine	Animal and human studies	↓ insulin levels, markers of insulin resistance, levels of triglycerides, uric acid, AST, ALT, and several markers of oxidative stress; ↑ GSH in animal studies Did not reduce postoperative complications or hepatic failure in human studies
[78]	Coenzyme Q10	Human studies	↓ MDA levels; ↑ SOD, GPX, and CAT levels
[81]	Dark chocolate	Human studies	↓ levels of sNOX2-dp, isoprostanes, and CK-18 M30 levels in NASH

CAT: catalase; GSH: glutathione; GSH-Px: glutathione peroxidase; MDA: malondialdehyde; MPO: myeloperoxidase; NAFLD: nonalcoholic fatty liver disease; NASH: nonalcoholic steatohepatitis; TG: triglyceride; TC: total cholesterol; sNOX2-dp: soluble NOX isoform 2; SOD: superoxide dismutase.

## Abbreviations

12/15 LOX: 12/15-Lipoxygenase
ACR: Acrolein
CAT: Catalase
cORP: Capacity oxidative reduction potential
COX-2: Cyclooxygenase-2
DAMP: Damage-associated molecular patterns
DBD: Donors after brain death
GCL: Glutamate cysteine ligase
GCLM: Modifier subunit of glutamate cysteine ligase
GM-CSF: Granulocyte-macrophage colony-stimulating factor
GPX4: Glutathione peroxidase 4
GSH: Glutathione
GSH-Px: Glutathione peroxidase
IL: Interleukin
iNOS: Inducible nitric oxide synthase
INF-*γ*: Interferon gamma
IPostC: Ischemic postconditioning
I/R: Ischemia/reperfusion
JNK: c-Jun N-terminal kinases
KC: Kupffer cells
MAPK: Mitogen-activated protein kinase
MCD: Methionine and choline deficient
MDA: Malondialdehyde
MSG: Monosodium L-glutamate
NAC: N-Acetyl-L-Cysteine
NADPH: Nicotinamide adenine dinucleotide phosphate
NAFLD: Nonalcoholic fatty liver disease
NASH: Nonalcoholic steatohepatitis
NF-*κ*B: Nuclear factor kappa-light-chain-enhancer of activated B cells
NO: Nitric oxide
NOX2: Nicotinamide adenine dinucleotide phosphate oxidase isoform 2
OLT: Orthotopic liver transplantation
PH: Partial hepatectomy
PI3K: Phosphatidylinositol 3-kinase
PRDX1: Peroxiredoxin 1
PRDX6: Peroxiredoxin 6
RCS: Reactive carbonyl species
RIPerC: Remote ischemic preconditioning
ROS: Reactive oxygen species
SOD: Superoxide dismutase
sORP: Static oxidative reduction potential
TC: Total cholesterol
TG: Triglycerides
TH: T helper cell
VIO: Vascular inflow occlusion.

## References

[1] Cederbaum A. I., Lu Y., Wu D. (2009). Role of oxidative stress in alcohol-induced liver injury. *Archives of Toxicology*.

[2] Ambade A., Mandrekar P. (2012). Oxidative stress and inflammation: essential partners in alcoholic liver disease. *International Journal of Hepatology*.

[3] Arias J.-L., Aller M. A., S??nchez-Patan F., Arias J. (2006). The inflammatory bases of hepatic encephalopathy. *European Journal of Gastroenterology & Hepatology*.

[4] Skowrońska M., Albrecht J. (2013). Oxidative and nitrosative stress in ammonia neurotoxicity. *Neurochemistry International*.

[5] Ucar F., Sezer S., Erdogan S., Akyol S., Armutcu F., Akyol O. (2013). The relationship between oxidative stress and nonalcoholic fatty liver disease: its effects on the development of nonalcoholic steatohepatitis. *Redox Report*.

[6] Podrini C., Borghesan M., Greco A., Pazienza V., Mazzoccoli G., Vinciguerra M. (2013). Redox homeostasis and epigenetics in non-alcoholic fatty liver disease (NAFLD). *Current Pharmaceutical Design*.

[7] Novo E., Parola M. (2012). The role of redox mechanisms in hepatic chronic wound healing and fibrogenesis. *Fibrogenesis & Tissue Repair*.

[8] Elias-Miró M., Jiménez-Castro M. B., Rodés J., Peralta C. (2013). Current knowledge on oxidative stress in hepatic ischemia/reperfusion. *Free Radical Research*.

[9] Li S., Tan H. Y., Wang N. (2015). The role of oxidative stress and antioxidants in liver diseases. *International Journal of Molecular Sciences*.

[10] Holmström K. M., Finkel T. (2014). Cellular mechanisms and physiological consequences of redox-dependent signalling. *Nature Reviews Molecular Cell Biology*.

[11] Cichoż-Lach H., Michalak A. (2014). Oxidative stress as a crucial factor in liver diseases. *World Journal of Gastroenterology*.

[12] Lee J., Giordano S., Zhang J. (2012). Autophagy, mitochondria and oxidative stress: cross-talk and redox signalling. *The Biochemical Journal*.

[13] Ajamieh H., Merino N., Candelario-Jalil E. (2002). Similar protective effect of ischaemic and ozone oxidative preconditionings in liver ischaemia/reperfusion injury. *Pharmacological Research*.

[14] Garcea G., Gescher A., Steward W., Dennison A., Berry D. (2006). Oxidative stress in humans following the Pringle manoeuvre. *Hepatobiliary & Pancreatic Diseases International*.

[15] Rosenfeldt F., Wilson M., Lee G. (2013). Oxidative stress in surgery in an ageing population: pathophysiology and therapy. *Experimental Gerontology*.

[16] Scolletta S., Carlucci F., Biagioli B. (2007). NT-proBNP changes, oxidative stress, and energy status of hypertrophic myocardium following ischemia/reperfusion injury. *Biomedicine & Pharmacotherapy*.

[17] Tsuchiya M., Sato E. F., Inoue M., Asada A. (2008). Open abdominal surgery increases intraoperative oxidative stress: can it be prevented?. *Anesthesia and Analgesia*.

[18] Braunwarth E., Stättner S., Fodor M. (2018). Surgical techniques and strategies for the treatment of primary liver tumours: hepatocellular and cholangiocellular carcinoma. *European Surgery*.

[19] Starlinger P., Pereyra D., Haegele S. (2018). Perioperative von Willebrand factor dynamics are associated with liver regeneration and predict outcome after liver resection. *Hepatology*.

[20] van Riel W. G., van Golen R. F., Reiniers M. J., Heger M., van Gulik T. M. (2016). How much ischemia can the liver tolerate during resection?. *Hepatobiliary Surgery and Nutrition*.

[21] Papadopoulos D., Siempis T., Theodorakou E., Tsoulfas G. (2013). Hepatic ischemia and reperfusion injury and trauma: current concepts. *Archives of Trauma Research*.

[22] Massip-Salcedo M., Roselló-Catafau J., Prieto J., Avíla M. A., Peralta C. (2007). The response of the hepatocyte to ischemia. *Liver International*.

[23] Alban F. T. E., Gyamfi D., van Golen R. F., Heger M., Patel V. B., Rajendram R., Preedy V. R. (2018). Reactive oxygen and nitrogen species and liver ischemia-reperfusion injury: an overview. *The Liver*.

[24] Zhang W., Wang M., Xie H. Y. (2007). Role of reactive oxygen species in mediating hepatic ischemia-reperfusion injury and its therapeutic applications in liver transplantation. *Transplantation Proceedings*.

[25] Hassan L., Bueno P., Ferrón-Celma I. (2005). Time course of antioxidant enzyme activities in liver transplant recipients. *Transplantation Proceedings*.

[26] Schwarz C., Fitschek F., Bar-Or D. (2017). Inflammatory response and oxidative stress during liver resection. *PLoS One*.

[27] Greten T. F., Papendorf F., Bleck J. S. (2005). Survival rate in patients with hepatocellular carcinoma: a retrospective analysis of 389 patients. *British Journal of Cancer*.

[28] Wang Z., Li Z., Ye Y., Xie L., Li W. (2016). Oxidative stress and liver cancer: etiology and therapeutic targets. *Oxidative Medicine and Cellular Longevity*.

[29] O’Leary D. P., Wang J. H., Cotter T. G., Redmond H. P. (2013). Less stress, more success? Oncological implications of surgery-induced oxidative stress. *Gut*.

[30] Sreevalsan S., Safe S. (2013). Reactive oxygen species and colorectal cancer. *Current Colorectal Cancer Reports*.

[31] Fernandez L., Carrasco-Chaumel E., Serafin A. (2004). Is ischemic preconditioning a useful strategy in steatotic liver transplantation?. *American Journal of Transplantation*.

[32] He N., Jia J. J., Li J. H. (2017). Remote ischemic perconditioning prevents liver transplantation-induced ischemia/reperfusion injury in rats: role of ROS/RNS and eNOS. *World Journal of Gastroenterology*.

[33] Hori T., Uemoto S., Chen F. (2014). Oxidative stress and extracellular matrices after hepatectomy and liver transplantation in rats. *World Journal of Hepatology*.

[34] Ngo B. T.-T., Beiras-Fernandez A., Hammer C., Thein E. (2013). Hyperacute rejection in the xenogenic transplanted rat liver is triggered by the complement system only in the presence of leukocytes and free radical species. *Xenotransplantation*.

[35] Schauer R. J., Bilzer M., Kalmuk S. (2001). Microcirculatory failure after rat liver transplantation is related to Kupffer cell-derived oxidant stress but not involved in early graft dysfunction. *Transplantation*.

[36] Tu Q., Xiong Y., Fan L. (2016). Peroxiredoxin 6 attenuates ischemia- and hypoxia-induced liver damage of brain-dead donors. *Molecular Medicine Reports*.

[37] Burke A., FitzGerald G. A., Lucey M. R. (2002). A prospective analysis of oxidative stress and liver transplantation. *Transplantation*.

[38] Corradini S. G., Micheletta F., Natoli S. (2005). High preoperative recipient plasma 7*β*-hydroxycholesterol is associated with initial poor graft function after liver transplantation. *Liver Transplantation*.

[39] Augusto V. S., Rodrigues A. J., Reis G. S. (2014). Evaluation of oxidative stress in the late postoperative stage of liver transplantation. *Transplantation Proceedings*.

[40] Madill J., Arendt B. M., Aghdassi E. (2009). Hepatic lipid peroxidation and antioxidant micronutrients in hepatitis virus C liver recipients with and without disease recurrence. *Transplantation Proceedings*.

[41] Riehle K. J., Haque J., McMahan R. S., Kavanagh T. J., Fausto N., Campbell J. S. (2013). Sustained glutathione deficiency interferes with the liver response to TNF-*α* and liver regeneration after partial hepatectomy in mice. *Journal of Liver: Disease & Transplantation*.

[42] Battal M., Çitgez B., Kartal A. (2015). Impact of GLP-1 analogue on oxidative damage and hepatic regeneration in experimental 70% hepatectomy model. *Hepato-Gastroenterology*.

[43] Beyer T. A., Xu W., Teupser D. (2008). Impaired liver regeneration in Nrf2 knockout mice: role of ROS-mediated insulin/IGF-1 resistance. *The EMBO Journal*.

[44] Florholmen-Kjær Å., Goll R., Fuskevåg O. M. (2016). The impact of partial hepatectomy on oxidative state in the liver remnant - an in vivo swine model. *Redox Biology*.

[45] Ben Mosbah I., Duval H., Mbatchi S. F. (2014). Intermittent selective clamping improves rat liver regeneration by attenuating oxidative and endoplasmic reticulum stress. *Cell Death & Disease*.

[46] Saito Y., Mori H., Takasu C. (2014). Beneficial effects of green tea catechin on massive hepatectomy model in rats. *Journal of Gastroenterology*.

[47] Drefs M., Thomas M. N., Guba M. (2017). Modulation of glutathione hemostasis by inhibition of 12/15-lipoxygenase prevents ROS-mediated cell death after hepatic ischemia and reperfusion. *Oxidative Medicine and Cellular Longevity*.

[48] Fausto N. (2001). Liver regeneration: from laboratory to clinic. *Liver Transplantation*.

[49] Haga S., Ozawa T., Yamada Y. (2014). p62/SQSTM1 plays a protective role in oxidative injury of steatotic liver in a mouse hepatectomy model. *Antioxidants & Redox Signaling*.

[50] Witort E., Capaccioli S., Becatti M. (2016). Albumin Cys34 adducted by acrolein as a marker of oxidative stress in ischemia-reperfusion injury during hepatectomy. *Free Radical Research*.

[51] Peh H. Y., Tan W. S. D., Liao W., Wong W. S. F. (2016). Vitamin E therapy beyond cancer: tocopherol versus tocotrienol. *Pharmacology & Therapeutics*.

[52] Zingg J.-M., Azzi A. (2004). Non-antioxidant activities of vitamin E. *Current Medicinal Chemistry*.

[53] Musso G., Gambino R., Cassader M. (2009). Non-alcoholic fatty liver disease from pathogenesis to management: an update. *Obesity Reviews*.

[54] Kamata H., Honda S.-I., Maeda S., Chang L., Hirata H., Karin M. (2005). Reactive oxygen species promote TNF*α*-induced death and sustained JNK activation by inhibiting MAP kinase phosphatases. *Cell*.

[55] Sakon S., Xue X., Takekawa M. (2003). NF-*κ*B inhibits TNF-induced accumulation of ROS that mediate prolonged MAPK activation and necrotic cell death. *The EMBO Journal*.

[56] Gambino R., Musso G., Cassader M. (2011). Redox balance in the pathogenesis of nonalcoholic fatty liver disease: mechanisms and therapeutic opportunities. *Antioxidants & Redox Signaling*.

[57] Phung N., Pera N., Farrell G., Leclercq I., Hou J. Y., George J. (2009). Pro-oxidant-mediated hepatic fibrosis and effects of antioxidant intervention in murine dietary steatohepatitis. *International Journal of Molecular Medicine*.

[58] Nan Y.-M., Wu W. J., Fu N. (2009). Antioxidants vitamin E and 1-aminobenzotriazole prevent experimental non-alcoholic steatohepatitis in mice. *Scandinavian Journal of Gastroenterology*.

[59] Foster T., Budoff M. J., Saab S., Ahmadi N., Gordon C., Guerci A. D. (2011). Atorvastatin and antioxidants for the treatment of nonalcoholic fatty liver disease: the St Francis Heart Study randomized clinical trial. *The American Journal of Gastroenterology*.

[60] Dufour J., Oneta C. M., Gonvers J. J. (2006). Randomized placebo-controlled trial of ursodeoxycholic acid with vitamin E in nonalcoholic steatohepatitis. *Clinical Gastroenterology and Hepatology*.

[61] European Association for the Study of the Liver (EASL) (2016). EASL–EASD–EASO clinical practice guidelines for the management of non-alcoholic fatty liver disease. *Journal of Hepatology*.

[62] Miller E. R., Pastor-Barriuso R., Dalal D., Riemersma R. A., Appel L. J., Guallar E. (2005). Meta-analysis: high-dosage vitamin E supplementation may increase all-cause mortality. *Annals of Internal Medicine*.

[63] Gerss J., Köpcke W. (2009). The questionable association of vitamin E supplementation and mortality--inconsistent results of different meta-analytic approaches. *Cellular and Molecular Biology*.

[64] Abner E. L., Schmitt F. A., Mendiondo M. S., Marcum J. L., Kryscio R. J. (2011). Vitamin E and all-cause mortality: a meta-analysis. *Current Aging Science*.

[65] Demiryilmaz I., Turan M. I., Kisaoglu A., Gulapoglu M., Yilmaz I., Suleyman H. (2014). Protective effect of nimesulide against hepatic ischemia/reperfusion injury in rats: effects on oxidant/antioxidants, DNA mutation and COX-1/COX-2 levels. *Pharmacological Reports*.

[66] Faghihzadeh F., Adibi P., Rafiei R., Hekmatdoost A. (2014). Resveratrol supplementation improves inflammatory biomarkers in patients with nonalcoholic fatty liver disease. *Nutrition Research*.

[67] Xu C., HaiYan Z., JianHong Z., Jing G. (2008). The pharmacological effect of polysaccharides from Lentinus edodes on the oxidative status and expression of VCAM-1mRNA of thoracic aorta endothelial cell in high-fat-diet rats. *Carbohydrate Polymers*.

[68] Qi H., Sun Y. (2015). Antioxidant activity of high sulfate content derivative of ulvan in hyperlipidemic rats. *International Journal of Biological Macromolecules*.

[69] Wang J., Cao Y., Wang C., Sun B. (2011). Wheat bran xylooligosaccharides improve blood lipid metabolism and antioxidant status in rats fed a high-fat diet. *Carbohydrate Polymers*.

[70] Mahmoud Y., Mahmoud A., Nassar G. (2015). Alpha-lipoic acid treatment of acetaminophen-induced rat liver damage. *Biotechnic & Histochemistry*.

[71] Salomone F., Li Volti G., Vitaglione P. (2014). Coffee enhances the expression of chaperones and antioxidant proteins in rats with nonalcoholic fatty liver disease. *Translational Research*.

[72] Corrao G., Zambon A., Bagnardi V., D’Amicis A., Klatsky A., Collaborative SIDECIR Group (2001). Coffee, caffeine, and the risk of liver cirrhosis. *Annals of Epidemiology*.

[73] Tverdal A., Skurtveit S. (2003). Coffee intake and mortality from liver cirrhosis. *Annals of Epidemiology*.

[74] Klatsky A. L., Morton C., Udaltsova N., Friedman G. D. (2006). Coffee, cirrhosis, and transaminase enzymes. *Archives of Internal Medicine*.

[75] Villagarcía H. G., Castro M. C., Arbelaez L. G. (2018). N -Acetyl- l -Cysteine treatment efficiently prevented pre-diabetes and inflamed-dysmetabolic liver development in hypothalamic obese rats. *Life Sciences*.

[76] Grendar J., Ouellet J. F., McKay A. (2016). Effect of N-acetylcysteine on liver recovery after resection: a randomized clinical trial. *Journal of Surgical Oncology*.

[77] Stewart B. W., Wild C. P. (2014). *World Cancer Report 2014*.

[78] Liu H.-T., Huang Y.-C., Cheng S.-B., Huang Y.-T., Lin P.-T. (2016). Effects of coenzyme Q10 supplementation on antioxidant capacity and inflammation in hepatocellular carcinoma patients after surgery: a randomized, placebo-controlled trial. *Nutrition Journal*.

[79] Del Ben M., Polimeni L., Carnevale R. (2014). NOX2-generated oxidative stress is associated with severity of ultrasound liver steatosis in patients with non-alcoholic fatty liver disease. *BMC Gastroenterology*.

[80] Carnevale R., Loffredo L., Pignatelli P. (2012). Dark chocolate inhibits platelet isoprostanes via NOX2 down-regulation in smokers. *Journal of Thrombosis and Haemostasis*.

[81] Loffredo L., del Ben M., Perri L. (2016). Effects of dark chocolate on NOX-2-generated oxidative stress in patients with non-alcoholic steatohepatitis. *Alimentary Pharmacology & Therapeutics*.

